# *Setaria viridis* Ethanol Extract Attenuates Muscle Loss and Body Fat Reduction in Sarcopenic Obesity by Regulating AMPK in High‐Fat Diet‐Induced Obese Mice

**DOI:** 10.1002/fsn3.70655

**Published:** 2025-07-18

**Authors:** Eun‐Young Kwon, Ji‐Won Kim, Ji‐Young Choi, Kyoungho Hwang, Youngji Han

**Affiliations:** ^1^ Department of Food Science and Nutrition Kyungpook National University Daegu Republic of Korea; ^2^ Center for Food and Nutritional Genomics Research Kyungpook National University Daegu Republic of Korea; ^3^ Center for Beautiful Aging Kyungpook National University Daegu Republic of Korea; ^4^ Department of Advanced Bioconvergence Kyungpook National University Daegu Republic of Korea; ^5^ Department of Food and Nutrition Chosun University Gwangju Republic of Korea; ^6^ Institute of Well‐Aging Medicare & CSU G‐LAMP Project Group Chosun University Gwangju Republic of Korea; ^7^ Graduate School of Artificial Intelligence Kyungpook National University Daegu Republic of Korea; ^8^ Bio‐Medical Research Institute Kyungpook National University Hospital Daegu Republic of Korea

## Abstract

Sarcopenic obesity, characterized by the concurrent presence of sarcopenia and obesity, presents a complex challenge due to the synergistic detrimental effects of muscle loss and excess body fat. This study investigated the potential of *Setaria viridis* (SV) supplementation for mitigating the impact of sarcopenic obesity on diet‐induced obese mice. Mice in the study were divided into groups and fed a normal diet, a high‐fat diet (HFD), or an HFD supplemented with 0.3% SV ethanol extract. After 16 weeks, SV supplementation significantly reduced body weight gain and white adipose tissue mass, improved plasma lipid profiles, and preserved liver function without affecting food intake. Additionally, SV enhanced skeletal muscle mass and hind‐limb thickness, and restored anabolic signaling pathways such as IGF‐1, PI3K/Akt, and mTOR. Notably, SV supplementation activated AMP‐activated protein kinase (AMPK), accompanied by increased expression of SIRT1 and PGC‐1α, leading to improved mitochondrial function and reduced intramuscular fat deposition. Mechanistically, 3D molecular docking simulations revealed that luteolin 7‐O‐glucoside, a SV‐derived flavonoid, binds to the AMPK ATP‐binding site with high affinity, providing structural insight into SV's AMPK‐activating potential. Furthermore, SV reduced levels of pro‐inflammatory cytokines (TNF‐α, IL‐6) and enhanced antioxidant enzyme activity (GSH, GR, GPx), thereby alleviating oxidative stress and tissue fibrosis. These findings suggest that SV supplementation counters obesity‐induced muscle atrophy and metabolic dysfunction through flavonoid‐mediated AMPK activation, supporting its potential as a safe and effective nutritional strategy for managing sarcopenic obesity.

## Graphical abstract

*Setaria viridis* (SV)‐derived flavonoids activate AMPK by binding to its ATP‐binding site, leading to enhanced SIRT1 activity and PGC‐1α expression. In high‐fat diet‐induced obese mice, SV supplementation increased muscle protein synthesis, reduced fat mass, and suppressed inflammation. These findings suggest AMPK as a key therapeutic target of SV in managing sarcopenic obesity.

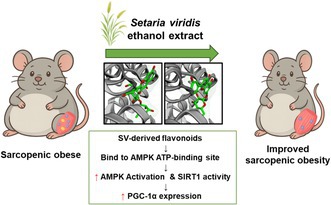

## 1 Introduction

Sarcopenic obesity, arising from the convergence of sarcopenia and obesity, presents significant health complexities (Zamboni et al. 2008). Sarcopenia, characterized by age‐related muscle loss, contrasts with obesity, which is marked by excessive body fat accumulation (Kalinkovich and Livshits 2017). Their coexistence synergistically heightens health risks and diminishes well‐being (Cauley 2015). This phenomenon poses diagnostic and treatment challenges, as conventional methods may inadequately address their combined effects (Xie, He, et al. 2021). Sarcopenic obesity escalates the incidence of mobility limitations, falls, and frailty while increasing the likelihood of metabolic disturbances, cardiovascular disease, and related complications (Salomon et al. 2012; Santos‐Eggimann et al. 2009; Scott et al. 2014; Shulman 2014). A comprehensive understanding of muscle loss and fat accumulation dynamics is vital for creating customized interventions. Effective strategies combining physical activity, nutrition, and lifestyle adjustments are critical for minimizing health risks and boosting quality of life.

Obesity can be managed via lifestyle modifications, including dietary adjustments, regular exercise, behavioral therapy, and medications, such as appetite suppressants and fat absorption inhibitors (Bischoff and Schweinlin 2020). Since obesity is a chronic condition, long‐term medication is often necessary. Sibutramine (an appetite suppressant) and orlistat (a fat absorption inhibitor) have been approved for extended use beyond 3 months in the Korean domestic market (Jeon et al. 2023). However, several of these medications primarily act centrally as appetite suppressants, leading to side effects such as headaches, vomiting, and diarrhea owing to fat absorption, raising concerns regarding misuse (Farr et al. 2016; Lean et al. 2014; Pi‐Sunyer et al. 2015). Therefore, research aimed at developing materials with high safety profiles and superior antiobesity efficacy to address the side effects associated with current antiobesity agents is imperative.

*Setaria viridis* (SV), classified under the *Setaria* genus within the Poaceae family, is a widespread and common annual grass native to Eurasia and North Africa. However, it has become naturalized in numerous other parts of the world, including East Asia (Fan et al. 2014). In Korea, SV has rarely been employed as a traditional remedy to stimulate urination by infusing and consuming the entire plant (Kwon et al. 2002). Aside from its application in treating various wounds, sores, eye congestion, and swelling, its extensive use in addressing other medical conditions has not been established, and its medicinal worth has not been widely acknowledged. Therefore, the current study examined the potential of SV as a dietary or supplementary modulator for metabolic disorders associated with obesity. Specifically, we explored the molecular mechanism through which SV exerts its antimetabolic syndrome effects in diet‐induced obese (DIO) mice.

## 2 Materials and Methods

### 2.1 Preparation of SV

The SV used in this experiment was obtained from Gyeongsan, Gyeongsangbuk‐do, South Korea, between August and September 2021. The aerial parts of SV (the entire plant) were dried, ground into powder, and subjected to extraction. A total of 100 g of dried powder was extracted with 10 volumes of 70% ethanol (v/v) at 60°C for 3 h using a reflux apparatus. The extraction procedure was repeated three times. Following extraction, the liquid was filtered using qualitative filter paper (Hyundai Micro, No. 22, 285 mm), vacuum‐concentrated under reduced pressure using a rotary evaporator (Eyela N‐1000, Tokyo Rikakikai Co. Ltd., Japan), and freeze‐dried (FD8512, IlShin BioBase, Korea). The final yield of the extract was 6.05%.

Metabolite profiling was performed using an Ultra Performance Liquid Chromatography system coupled with a Quadrupole Time‐of‐Flight Mass Spectrometer (UPLC‐QTOF‐MS; negative ion mode). Chromatographic separation was achieved using a reverse‐phase C18 column (100 × 2.1 mm, 1.7 μm). The mobile phases consisted of 0.1% formic acid in water (A) and acetonitrile (B), with a gradient elution. The flow rate was set at 0.3 mL/min, and the column temperature was maintained at 40°C. Injection volume was 2 μL.

The compounds kaempferol 3‐O‐neohesperidoside and luteolin 7‐O‐glucoside were identified based on their retention times (RT 4.82 and 4.96 min, respectively) and mass spectral features (Figure S1).

### 2.2 Experimental Animals and Diet

Male C57BL/6J mice (*n* = 24, 4‐week‐old; Jackson Laboratory, Bar Harbor, ME, USA) were used in this study. Only male mice were selected to minimize physiological variability due to the estrous cycle in females, which can influence metabolic and hormonal responses (Lovejoy, Sainsbury, and Group 2009). Upon arrival, the mice were placed in the same cage, under a 12‐h light–dark cycle and a stable temperature of 22°C ± 2°C. In the first week, they were fed a standard chow diet. All mice were maintained in a room with controlled temperature (20°C–23°C) and lighting (alternating 12‐h light and dark periods) and fed a commercial nonpurified diet for 1 week after arrival. Subsequently, the mice were randomly assigned to three groups (*n* = 8). They were respectively fed the following experimental diets for 20 weeks (Table S1): standard diet control (ND), high‐fat diet control (HFD, 60% of kcal from fat), and HFD with 0.3% SV extract (SV, HFD with 0.3% SV ethanol extract, w/w). The dietary dose of *Setaria viridis* extract (0.3%, w/w) was determined based on preliminary in vitro experiments using 3 T3‐L1 preadipocytes, where significant antiadipogenic effects were observed at concentrations ≥ 100 μg/mL. Assuming an average food intake of 3.5 g/day for a 25 g mouse, this dietary concentration corresponds to approximately 10.5 mg/day, or ~420 mg/kg/day. With an estimated oral bioavailability of 10%, systemic exposure was calculated to be approximately 42 mg/kg/day, which is within the effective range observed in vitro.

The mice had ad libitum access to food and distilled water throughout the experimental period. Food intake was recorded daily, and body weight was monitored biweekly. The animal experiments in this study were conducted in accordance with the ethical standards of the Institutional Animal Care and Use Committee (IACUC) of Kyungpook National University, and the protocol was approved on August 24, 2020 (Approval No. KNU‐2020‐0090). The study also adhered to the principles outlined in the ARRIVE (Animal Research: Reporting of In Vivo Experiments) guidelines (Nam et al. 2018).

### 2.3 Left Hind Leg Thickness

The thickness of the left hind leg was measured every 4 weeks during the experiment using a Blutec digital caliper (BD500; BLUETEC, Seoul, South Korea).

### 2.4 Histopathological and Immunohistochemical Analysis

Epididymal white adipose tissue (eWAT), liver, and gastrocnemius muscle tissues were collected and fixed in 10% formalin. The eWAT and liver tissue were processed for paraffin embedding, sectioned, and stained with hematoxylin and eosin and Masson's trichrome, followed by microscopic observation (Nikon, Tokyo, Japan) at ×200 magnification (Fischer et al. 2008; Foot 1933). Similarly, gastrocnemius muscle tissue was sectioned, stained with H&E and Sirius red, and subjected to immunohistochemistry, IGF‐1, and myostatin following established protocols (Kim et al. 2022a). Fiber cross‐sectional and collagen areas were analyzed using ImageJ (Encarnacion‐Rivera et al. 2020).

### 2.5 Western Blot Analysis

Protein quantification in the cytoplasm and membrane was performed using the Bradford method (Bradford 1976). Proteins were separated on a 10% sodium dodecyl sulfate‐polyacrylamide gel and subjected to electrophoresis in Tris‐glycine buffer for 1 h. Membranes were then blocked with 5% skim milk in Tris‐buffered saline (TBS) containing 0.1% Tween‐20 for 60 min at room temperature, followed by overnight incubation with primary antibodies at 4°. After washing, the membranes were incubated for 30 min in TBST buffer with secondary antibodies (anti‐rabbit IgG, Amersham, UK, or anti‐goat IgG, Abcam, USA) for 1 h at room temperature. Membranes were washed in TBST for 30 min, and immunoreactive bands were visualized using an enhanced chemiluminescence kit (Pierce Chemical Co., IL, USA). The molecular weights of the bands were confirmed. A list of antibodies is provided in Table S2.

### 2.6 mRNA Expression Analysis

The procedure for mRNA extraction followed the method outlined in the previous study. (Han et al. 2020). Total mRNA was converted to cDNA using the QuantiTect Reverse Transcription Kit (Qiagen, Hilden, Germany). The expression levels of mRNA were then measured through real‐time quantitative polymerase chain reaction (PCR) analysis utilizing the QuantiTect SYBR Green PCR Kit (Qiagen) and SDS7000 sequence detection system (Applied Biosystems, Foster City, CA, USA).

### 2.7 Plasma Inflammatory Cytokines

The levels of plasma inflammatory cytokines, interferon‐gamma (IFN‐γ), tumor necrosis factor (TNF‐α), interleukin one beta (IL‐1β), and interleukin 6 (IL‐6) were assessed by a commercial kit (Bio‐Rad Laboratories Inc., Hercules, CA, USA). The analysis used the Luminex 200 LabMAP system (Luminex, Austin, TX, USA) and Bio‐Plex Manager software (version 4.1.1; Bio‐Rad Laboratories Inc.).

### 2.8 Antioxidant Enzyme Activity

Glutathione reductase (GR) activity was assessed by monitoring the oxidation of nicotinamide adenine dinucleotide phosphate following the protocol of Pinto and Bartley (Pinto and Bartley 1969). The total glutathione (GSH) content was measured using Ellman's method (Owens and Belcher 1965).

### 2.9 Lipid Peroxide Content

The lipid peroxide content in erythrocytes was determined using the method described by Tarladgis et al. (Tarladgis et al. 1964). The concentration of 2‐thiobarbituric acid reactive substances (TBARS) was calculated using the extinction coefficient of malondialdehyde.

### 2.10 Statistical Analysis

Data are expressed as mean ± standard error of the mean (SEM). Statistical analyses were conducted using SPSS software (version 27; SPSS, Chicago, IL, USA). A one‐way analysis of variance (ANOVA) was used to compare variables among experimental groups, followed by Tukey's post hoc test to assess significant differences (*p* < 0.05) between groups.

### 2.11 Molecular Docking

To investigate the potential interaction between AMP‐activated protein kinase (AMPK) and plant‐derived flavonoids, molecular docking simulations were performed using AutoDock Vina. The AMPK crystal structure (PDB ID: 4CFE) was retrieved from the Protein Data Bank and prepared using AutoDockTools (MGLTools v1.5.7) by removing water molecules and adding polar hydrogens and Gasteiger charges. The ATP‐binding site was defined based on the location of the co‐crystallized AMP molecule.

Ligands Kaempferol 3‐O‐neohesperidoside and Luteolin 7‐O‐glucoside were obtained from the PubChem database in .sdf format and converted to .pdbqt format using Open Babel with energy minimization and 3D coordinate generation. Docking simulations were conducted using a grid box centered at *x* = 563.36, *y* = 93.73, *z* = 1019.73, with dimensions of 20 × 20 × 20 Å, encompassing the ATP‐binding pocket.

Docking was executed with the default exhaustiveness parameter. The top‐scoring poses were ranked by binding affinity, and the best pose was visualized using py3Dmol for spatial analysis.

## 3 Results

### 3.1 SV Supplementation Attenuates Adiposity and Dyslipidemia Without Inducing Hepatic Damage in DIO Mice

**FIGURE 1:**
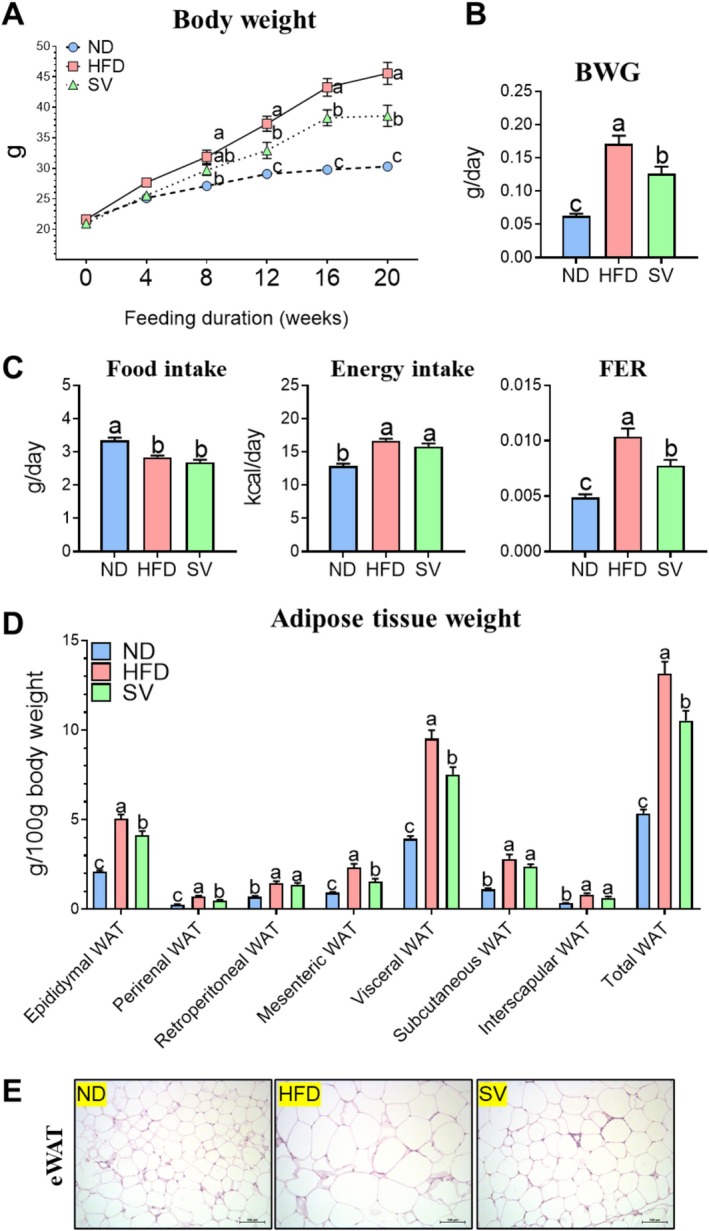
Effects of 20‐week SV supplementation on (A) body weight, (B) body weight gain, (C) food and energy intake, the food efficiency ratio, (D) adipose tissue weight, and (E) H&E staining of epididymal white adipose tissue. Data are presented as the mean ± SEM. Different superscript letters (a, b, and c) indicate significant differences among the groups (*p* < 0.05). BWG, body weight gain; eWAT, epididymal white adipose tissue; food efficiency ratio; HFD, high‐fat diet (20% kcal from fat); ND, normal diet (5% kcal from fat); SV, HFD with 0.3% SV extract; WAT, white adipose tissue. H&E images were captured at 200× magnification with a scale bar of 100 μm.

SV is a prevalent and significant weed in various regions worldwide, particularly in agricultural fields, gardens, and disturbed areas. As a member of the Gramineae family, it is recognized as a traditional medicinal herb in China. It has been historically used for acute eczema, warts, and corns and to relieve abdominal distension, belching, and diarrhea (Li et al. 2016). Research has revealed that SV contains a high concentration of polyphenols, tannins, and sugars, contributing to its potent, powerful effects in reducing oxidative stress, inflammation, allergic reactions, and itching (Cheng et al. 2013). However, its efficacy in HFD‐fed mice has previously not been evaluated. This study is the first to explore SV's impact on sarcopenic obesity. Supplementation with SV significantly reduced body weight and weight gain compared with HFD feeding without SV supplementation, as depicted in Figure 1A,B. Interestingly, there were no significant differences in food and energy intake between the HFD and SV groups. However, the food efficiency ratio (FER) was significantly lower in the SV group than in the HFD group, as demonstrated in Figure 1C. The observed decline in the FER, without alterations in food intake, suggests that SV supplementation does not affect appetite regulation but effectively promotes weight loss. Moreover, SV supplementation significantly decreased the weight of various WATs compared with HFD feeding without SV supplementation, as illustrated in Figure 1D. Morphological analyses indicate that the HFD group exhibited larger lipid accumulation areas in the eWAT than the ND group, according to Figure 1E. These findings suggest that SV‐induced weight loss primarily results from reduced body fat.

In addition to reductions in adiposity, SV supplementation elicited favorable alterations in systemic lipid metabolism. As shown in Table 1, compared with the HFD group, SV‐treated mice exhibited significantly lower serum concentrations of free fatty acids and triglycerides, reflecting improved lipid handling. Total cholesterol and non‐HDL cholesterol levels were also markedly reduced following SV administration, whereas HDL cholesterol remained elevated. Although the HDL‐to‐total cholesterol ratio (HTR) and atherogenic index (AI) did not differ significantly among groups, the SV group demonstrated a significant reduction in the Apo B/Apo A1 ratio, suggesting an improved lipoprotein profile and a potential decrease in cardiovascular risk.

**TABLE 1:** Effects of 20‐week SV supplementation on plasma lipid profiles and liver enzyme levels in C57BL/6J mice fed a high‐fat diet.

	ND	HFD	SV
Free fatty acid (mol/L)	1.10 ± 0.04^a^	1.08 ± 0.02^a^	0.94 ± 0.02^b^
Triglyceride (mmol/L)	0.90 ± 0.08^a^	1.03 ± 0.08^a^	0.78 ± 0.02^b^
Total cholesterol (mmol/L)	4.15 ± 0.27^c^	7.26 ± 0.59^a^	5.46 ± 0.33^b^
HDL cholesterol (mmol/L)	0.97 ± 0.05^b^	1.62 ± 0.08^a^	1.40 ± 0.08^a^
Non‐HDL cholesterol (mmol/L)	3.18 ± 0.0.23^c^	5.64 ± 0.53^a^	4.06 ± 0.29^b^
HTR (%)	23.64 ± 0.87	22.81 ± 1.35	25.88 ± 1.24
AI	3.28 ± 0.16	3.46 ± 0.23	2.93 ± 0.19
Apo A1 (mg/ml)	0.28 ± 0.02^b^	0.36 ± 0.01^a^	0.35 ± 0.01^a^
Apo B (mg/ml)	0.21 ± 0.01^b^	0.39 ± 0.05^a^	0.26 ± 0.02^b^
Apo B/Apo A1	0.77 ± 0.05^a^	1.09 ± 0.10^a^	0.73 ± 0.05^b^
AST (Karman/mL)	53.50 ± 6.85^c^	179.26 ± 23.86^a^	96.9 ± 14.41^b^
ALT (Karman/mL)	3.15 ± 0.74^c^	166.65 ± 30.09^a^	73.69 ± 16.81^b^

*Note:* Data are mean ± S.D. Different superscript letters (a, b, and c) indicate significant differences among the groups (*p* < 0.05).

Abbreviations: AI2, atherogenic index = [(Total‐C) – (HDL‐C)]/HDL‐C; ApoA‐I, apolipoprotein A‐I; Apo B, apolipoprotein B; FFA, free fatty acid; HFD, high‐fat diet (20% kcal from fat); HTR1, (HDL‐C/Total‐C) × 100; ND, normal diet (5% kcal from fat); non‐HDL‐C, (Total‐C) – (HDL‐C); SV, HFD with 0.3% SV extract.

SV supplementation also conferred hepatoprotective effects. As expected, HFD feeding led to pronounced elevations in serum aspartate aminotransferase (AST) and alanine aminotransferase (ALT) levels, indicative of hepatic injury. These increases were significantly attenuated in the SV group, suggesting that SV mitigates HFD‐induced liver damage. While liver enzyme levels in the SV group did not fully normalize to those of the ND group, the partial reversal nonetheless supports the hepatoprotective potential of SV extract.

Collectively, these findings indicate that SV supplementation not only attenuates HFD‐induced weight gain and adipose tissue expansion but also improves systemic lipid profiles and mitigates hepatic dysfunction. Notably, these effects were observed without changes in caloric intake. These observations underscore the therapeutic potential of *Setaria viridis* in the context of obesity and associated metabolic disturbances.

Taken together, SV supplementation in HFD‐fed mice resulted in significant reductions in adiposity and improvements in lipid profiles, without inducing hepatotoxicity. These outcomes suggest that SV may serve as a safe and effective dietary strategy for managing obesity and its metabolic complications.

### 3.2 SV Supplementation Promoted Muscle Growth and Increased Leg Thickness in DIO Mice

**FIGURE 2:**
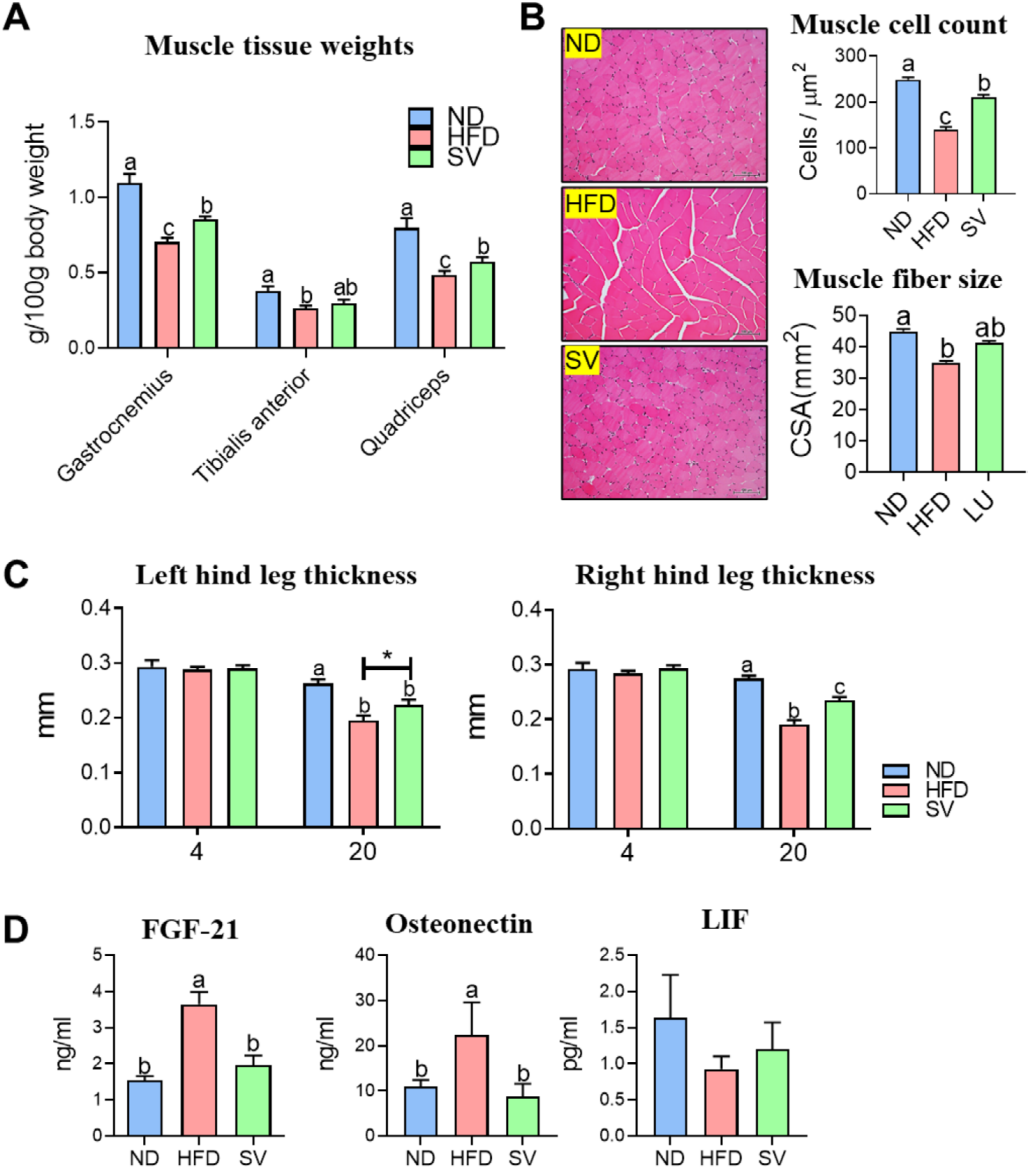
Effects of 20‐week SV supplementation on (A) muscle tissue weight, (B) H&E staining of the gastrocnemius, (C) leg thickness, and (D) levels of plasma myogenic cytokines. Data are presented as the mean ± SEM. Different superscript letters (a, b, and c) indicate significant differences among the groups (*p* < 0.05). FGF, fibroblast growth factor 21; HFD, high‐fat diet (20% kcal from fat); LIF, leukemia inhibitory factor; ND, normal diet (5% kcal from fat); SV, HFD with 0.3% SV extract. H&E images were captured at 200× magnification with a scale bar of 100 μm.

Numerous studies have suggested that both short‐term and long‐term intake of an HFD significantly increases body weight and adipose tissue weight, resulting in decreased grip strength and a reduction in muscle mass (Lee et al. 2015; Roseno et al. 2015; Xie, He, et al. 2021). The findings presented in Figure 2 have significant implications for understanding the impact of HFD feeding and SV supplementation on skeletal muscle health. Skeletal muscle weight was measured, encompassing the gastrocnemius, tibialis anterior, and quadriceps (Figure 2A). Consistent with previous results, muscle tissue weights significantly lowered in the HFD group compared to the ND group. This reduction in muscle mass, particularly noticeable in key muscle groups, is attributable to the metabolic challenges posed by excessive fat intake, which potentially leads to anabolic or catabolic conditions in muscle tissues (Herrenbruck and Bollinger 2019; Pauly et al. 2014). However, SV supplementation significantly increased muscle weight compared with HFD feeding without SV supplementation. Additionally, H&E staining‐based morphological analysis of muscle tissue revealed a significant decrease in muscle fiber number and size in the HFD group compared with the ND group (Figure 2B). Conversely, the SV group exhibited significant increases in both muscle fiber number and size compared to the HFD group. Moreover, hind‐leg thickness, which could be regarded as a diagnostic factor for sarcopenia (Perkisas et al. 2021), was significantly greater in the SV group than in the HFD group. This increase in hind‐leg thickness observed in the SV group compared to the HFD group suggests enhanced muscle functionality, possibly translating into superior physical performance and mobility (Figure 2C). This aspect is crucial for developing dietary interventions aimed at populations at risk of sarcopenic obesity, a condition defined by the simultaneous presence of obesity and the decline in muscle mass and functionality. Taken together, it seems to have a protective or potentially restorative impact on muscle mass. SV‐treated mice displayed a significant increase in muscle weight and enhancement in muscle fiber dimensions relative to their HFD counterparts. This suggests that SV supplementation may counteract some of the adverse effects of an HFD on muscle tissues.

### 3.3 SV Supplementation Enhanced Protein Synthesis and Mitigated Protein Degradation in DIO Mice Muscle

**FIGURE 3:**
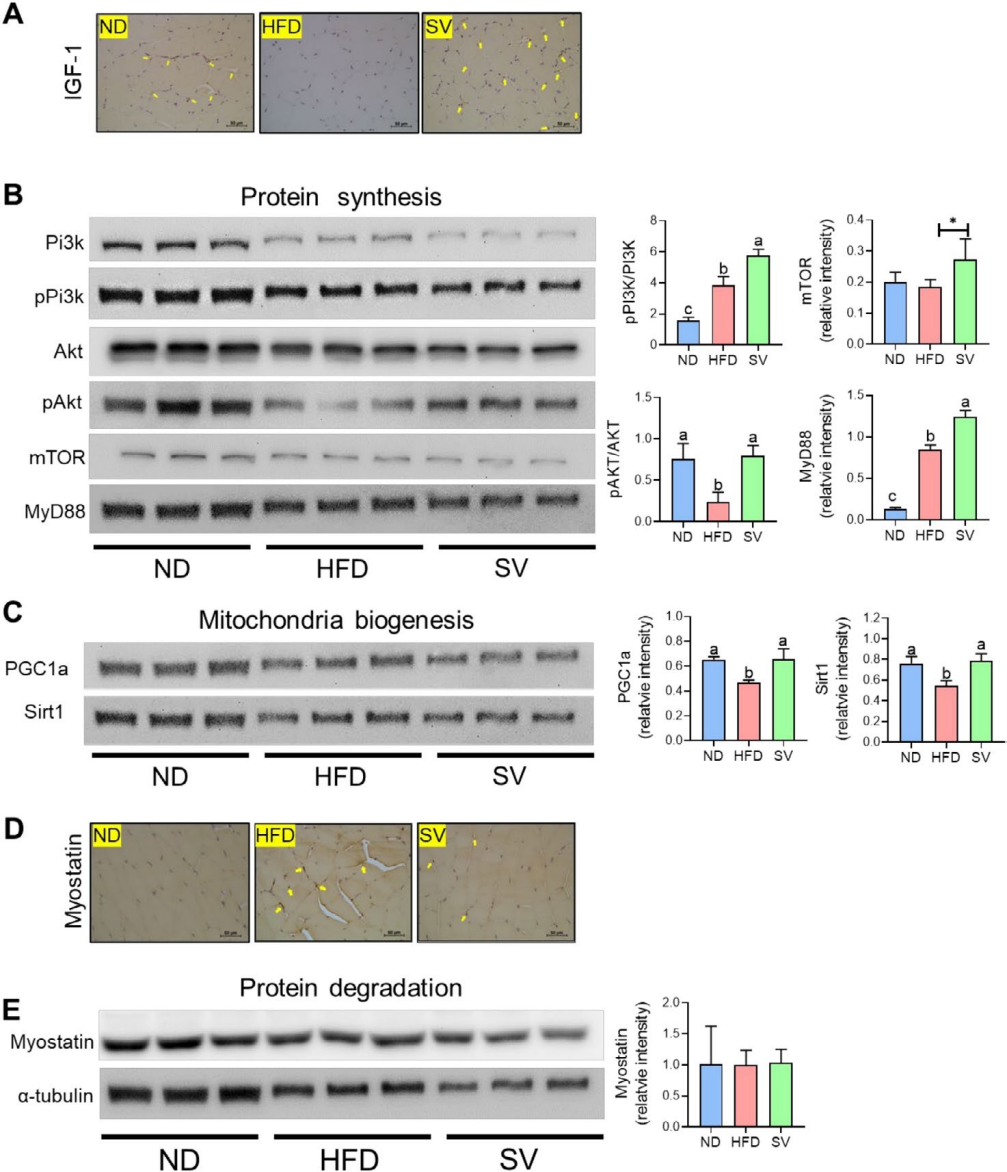
Effects of 20‐week SV supplementation on (A) IGF‐1 expression, (B) the protein expression levels of the PI3K–Akt–mTOR signaling pathway, (C) PGC1 α, Sirt1, (D) myostatin, and (E) its protein and mRNA expression levels. Data are presented as the mean ± SEM. Different superscript letters (a, b, and c) indicate significant differences among the groups (*p* < 0.05). HFD, high‐fat diet (20% kcal from fat); IGF‐1, insulin‐like growth factor 1; mTOR, mammalian target of rapamycin; MyD88, myeloid differentiation primary response 88; ND, normal diet (5% kcal from fat); PGC1α, peroxisome proliferator‐activated receptor gamma coactivator 1‐alpha; Pi3k, phosphoinositide 3‐kinase; Sirt1, sirtuin 1; SV, HFD with 0.3% SV extract. IHC‐stained images were captured at 200× magnification, with a scale bar representing 100 μm.

Insulin resistance is a pathological condition wherein cells exhibit a reduced responsiveness to insulin, resulting in impaired glucose uptake and elevated blood glucose levels. This condition is exacerbated in obesity, where chronic inflammation plays a pivotal role in inducing insulin resistance (Kim et al. 2000; Lee et al. 2011). IGF‐1 is a hormone structurally similar to insulin and is predominantly synthesized in the liver following stimulation by the growth hormone. It is essential for protein synthesis, primarily by activating the phosphoinositide 3‐kinase (PI3K)/protein kinase B (Akt) signaling pathway (Bibollet‐Bahena and Almazan 2009). As depicted in Figure 3A, IGF‐1 levels significantly lowered in the HFD group compared with those in the ND group. However, SV supplementation (likely referring to a specific supplement or intervention) ameliorated this decline in gastrocnemius muscle tissue. Furthermore, the expression of IGF‐1 downstream signaling components, such as PI3K, Akt, and mTOR, was markedly reduced in the HFD group compared with the ND group, as illustrated in Figure 3B. This reduction possibly reflects the detrimental effects of HFD‐induced obesity on the growth factor environment owing to insulin resistance. Conversely, SV supplementation mitigated the decline in IGF‐1 and its downstream factor expressions observed in the HFD group. These findings suggest that SV supplementation can either directly or indirectly enhance the signaling pathway crucial for muscle growth and maintenance, even under conditions of HFD‐induced insulin resistance. Additionally, the SV group significantly upregulated the protein expression of peroxisome proliferator‐activated receptor gamma coactivator 1‐alpha (PGC1α) and sirtuin 1 (Sirt1) compared with the HFD group, both of which are downstream targets of AMPK involved in mitochondrial biogenesis and lipid metabolism (Figure 3C). Immunohistochemical analysis revealed a trend toward suppressed myostatin expression in the gastrocnemius of the SV group (Figure 3D), despite the lack of significant differences in myostatin protein or mRNA expression across the experimental groups (Figure 3E). Overall, these results underscore dietary SV supplementation's potential therapeutic role in managing HFD‐induced muscular changes. In sarcopenic obesity, SV supplementation potentially offers a strategy toward counteracting HFD feeding's negative effects on muscle growth regulators, thereby supporting muscle health and preventing the decline typically associated with such diets.

### 3.4 SV Supplementation Ameliorated Muscle Lipid Infiltration in DIO Mice

**FIGURE 4:**
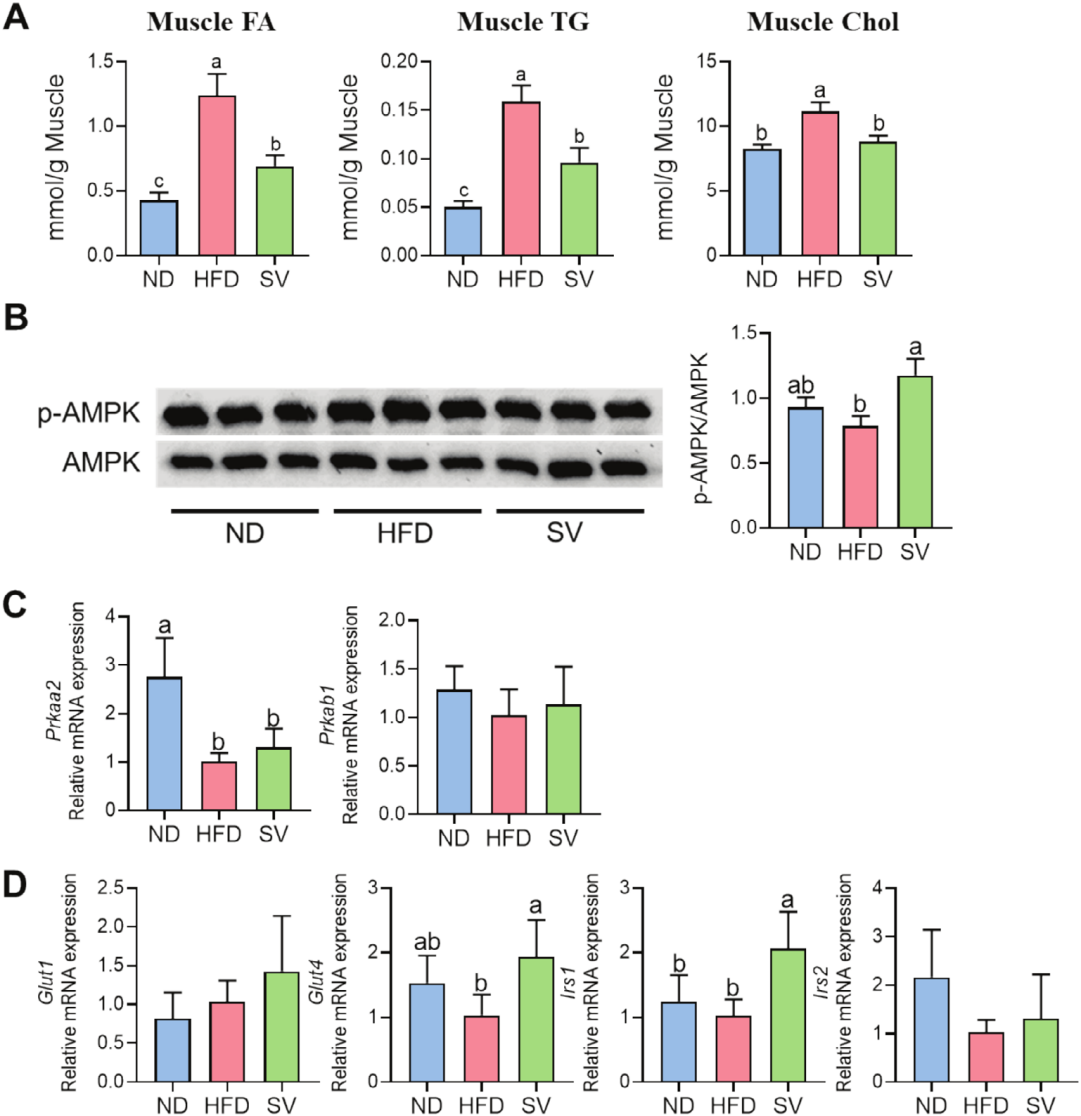
Effects of 20‐week SV supplementation on (A) muscle lipid content, (B) the protein expression levels of the AMPK signaling pathway, and (C) its mRNA expression levels. (D) Predicted binding poses of kaempferol 3‐O‐neohesperidoside and luteolin 7‐O‐glucoside major flavonoids enriched in *Setaria viridis* —within the ATP‐binding pocket of AMPK. Data are presented as the mean ± SEM. Different superscript letters (a, b, and c) indicate significant differences among the groups (*p* < 0.05). AMPK, AMP‐activated protein kinase; HFD, high‐fat diet (20% kcal from fat); ND, normal diet (5% kcal from fat); Prkaa2, 5′‐AMP‐activated protein kinase catalytic subunit alpha‐2; Prkab1, 5′‐AMP‐activated protein kinase catalytic subunit beta‐1; SV, HFD with 0.3% SV extract. Representative 3D binding poses of kaempferol 3‐O‐neohesperidoside (left) and luteolin 7‐O‐glucoside and luteolin (right) are shown in the ATP‐binding pocket of AMPK (PDB ID: 4CFE). The protein is rendered in a ribbon format (gray), and the ligands are depicted as green stick models with red oxygen atoms.

Muscle lipid infiltration, also known as intramuscular fat accumulation, represents a significant physiological and pathological phenomenon, and it is a characteristic phenotype of sarcopenic obesity (Li, Yu, et al. 2022; Song et al. 2004). Appropriate lipid accumulation is not inherently detrimental and can, under normal physiological conditions, provide a readily available energy source during prolonged or intense physical activity (Badin et al. 2013). However, excessive lipid infiltration in muscle potentially leads to muscle insulin resistance, impaired muscle function, and altered metabolic homeostasis, contributing to the development of metabolic disorders (Kim et al. 2022b). As shown in Figure 4A, muscle lipid content was significantly increased in the HFD group compared to the ND group, while SV supplementation reduced the muscle lipid content relative to HFD feeding without SV supplementation. In sarcopenic obesity, excessive fat deposits, not only in adipose tissue but also intramuscularly, disrupt normal muscle function. Adenosine 5′‐monophosphate‐activated protein kinase (AMPK) activation induces modulated adipose tissue metabolism, facilitating lipid turnover and reducing overall body fat, thus addressing the obesity component of the condition (Hardie 2011). As shown in Figure 4B,C, SV supplementation activated AMPK and enhanced its mRNA expression compared with HFD feeding without SV supplementation. To further elucidate the potential molecular basis of AMPK activation by SV, molecular docking analyses were performed using two representative flavonoid glycosides: luteolin 7‐O‐glucoside and kaempferol 3‐O‐neohesperidoside (Figure 4D, Table S3). The results revealed that luteolin exhibited a stronger binding affinity to AMPK (−7.2 kcal/mol) compared to kaempferol (−4.3 kcal/mol), suggesting a greater potential for direct molecular interaction with the AMP‐binding site. While kaempferol generated four docking poses with a well‐defined top‐ranked conformation (RMSD = 0.000 Å), luteolin yielded nine energetically favorable poses, indicating higher conformational flexibility and interaction stability. Although direct biochemical validation is required, the broader and more stable binding profile of luteolin supports its role as a principal AMPK‐targeting component of SV. Previous studies have shown that luteolin and its glycosides activate AMPK and enhance glucose and lipid metabolism in skeletal muscle (Li, Xu, et al. 2022), whereas kaempferol's effects appear more modest or context‐dependent (Moore et al. 2023). Collectively, these findings suggest that luteolin glycosides may contribute more substantially to the AMPK‐mediated metabolic improvements observed with SV supplementation, providing a mechanistic basis for their antiobesity and antisarcopenic effects.

### 3.5 SV Supplementation Mitigated the Inflammatory Response in DIO Mice

**FIGURE 5:**
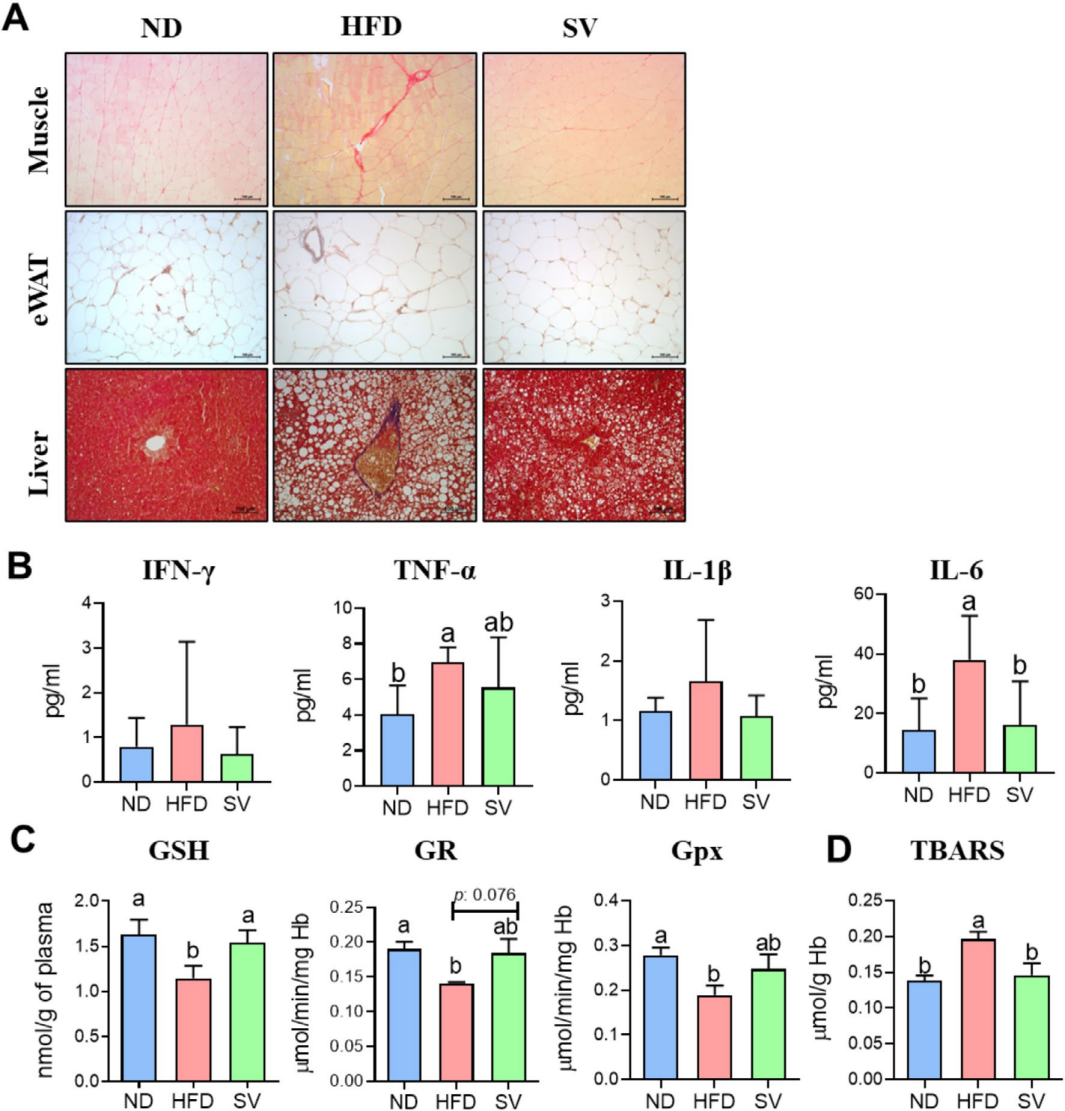
Effects of 20‐week SV supplementation on (A) fibrosis in muscle tissue, eWAT, and liver tissue; (B) plasma inflammatory cytokines; (C) GSH and its enzyme activities; and (D) TBARS content. Data are presented as the mean ± SEM. Different superscript letters (a, b) indicate significant differences among the groups (*p* < 0.05). GPX, glutathione peroxidase; GR, glutathione reductase; GSH, glutathione; HFD, high‐fat diet (20% kcal from fat); IFN‐γ, interferon gamma; IL, interleukin; ND, normal diet (5% kcal from fat); SV, HFD with 0.3% SV extract; TBARS, thiobarbituric acid reactive substances; TNF‐α, tumor necrosis factor. H&E and Masson's trichrome‐stained images were captured at 200× magnification, with a scale bar representing 100 μm.

Muscle fibrosis is marked by the excessive buildup of extracellular matrix components, especially collagen, resulting in the stiffening and decline of function in muscle tissue (Ueha et al. 2012). This issue is often a consequence of chronic injury, inflammation, or degenerative diseases such as muscular dystrophy (Smith and Barton 2018). Obesity is closely linked to chronic inflammation induced by adipocyte hypertrophy, which significantly contributes to the development of metabolic complications via elevated levels of pro‐inflammatory cytokines (Engin 2017). As illustrated in Figure 5A, histological analysis revealed fibrosis in muscle, adipose, and hepatic tissues in the HFD group. We subsequently measured the plasma concentrations of inflammatory cytokines (IFN‐γ, TNF‐α, IL‐1β, and IL‐6). TNF‐α exhibited a decreasing trend in the SV group compared to the HFD group. IL‐6 concentrations significantly decreased in the SV group compared to the HFD group.

Furthermore, in HFD‐induced obesity, increased fat oxidation potentially elevates the reactive oxygen species production, contributing to chronic inflammation (Kesh et al. 2016). In our study, SV supplementation significantly increased GSH levels and tended to increase GR and glutathione peroxidase activities. Consistent with these results, the TBARS content also significantly decreased with SV supplementation. Therefore, SV supplementation can inhibit fibrosis in muscle tissue and other tissues by alleviating obesity‐induced chronic inflammation through the enhancement of antioxidant function. This suggests that SV supplementation not only reduces pro‐inflammatory cytokine levels but also increases antioxidant enzyme activity, collectively mitigating oxidative stress and chronic inflammation. Consequently, this reduction in inflammation and oxidative stress potentially prevents or mitigates fibrotic changes in various tissues, ultimately improving overall tissue health and function in individuals with obesity.

Our findings suggested that SV supplementation ameliorated obesity‐associated metabolic dysfunction, muscle atrophy, and chronic inflammation in HFD‐fed mice. These effects were mediated, at least in part, through AMPK activation and modulation of lipid and protein metabolism. Given its favorable safety profile and bioactive composition, SV represents a promising candidate for dietary intervention strategies targeting sarcopenic obesity and metabolic syndrome. Despite these promising results, several limitations should be acknowledged. First, SV supplementation was administered at a single dose and for a fixed duration; thus, dose–response relationships and long‐term safety remain to be determined. Second, although UPLC analysis confirmed the presence of flavonoid glycosides such as luteolin 7‐O‐glucoside and kaempferol 3‐O‐neohesperidoside, their relative or absolute concentrations were not quantitatively analyzed in a manner that would allow precise estimation of their biological contributions. Without a detailed quantification and bioavailability assessment, it remains challenging to determine the extent to which these individual compounds drive the observed physiological effects. Third, while molecular docking provided valuable insight into potential AMPK–ligand interactions, it is an in silico tool that cannot substitute for experimental validation of biological activity. Nevertheless, this study possesses several notable strengths. It is the first to comprehensively investigate the antiobesity and antisarcopenic effects of SV in a high‐fat diet‐induced obesity model, integrating physiological, biochemical, histological, and molecular analyses. The findings provide mechanistic insight into the role of AMPK signaling in mediating the metabolic benefits of SV, supported by both in vivo evidence and in silico molecular docking simulations. Moreover, the use of a polyphenol‐rich natural extract with confirmed bioactive constituents offers translational potential for the development of food‐based interventions targeting obesity‐related muscle dysfunction. By highlighting the dual effects of SV on both adiposity and muscle preservation, this study lays the foundation for further research into plant‐derived therapeutic strategies for sarcopenic obesity and metabolic disorders.

## 4 Conclusion

**FIGURE 6:**
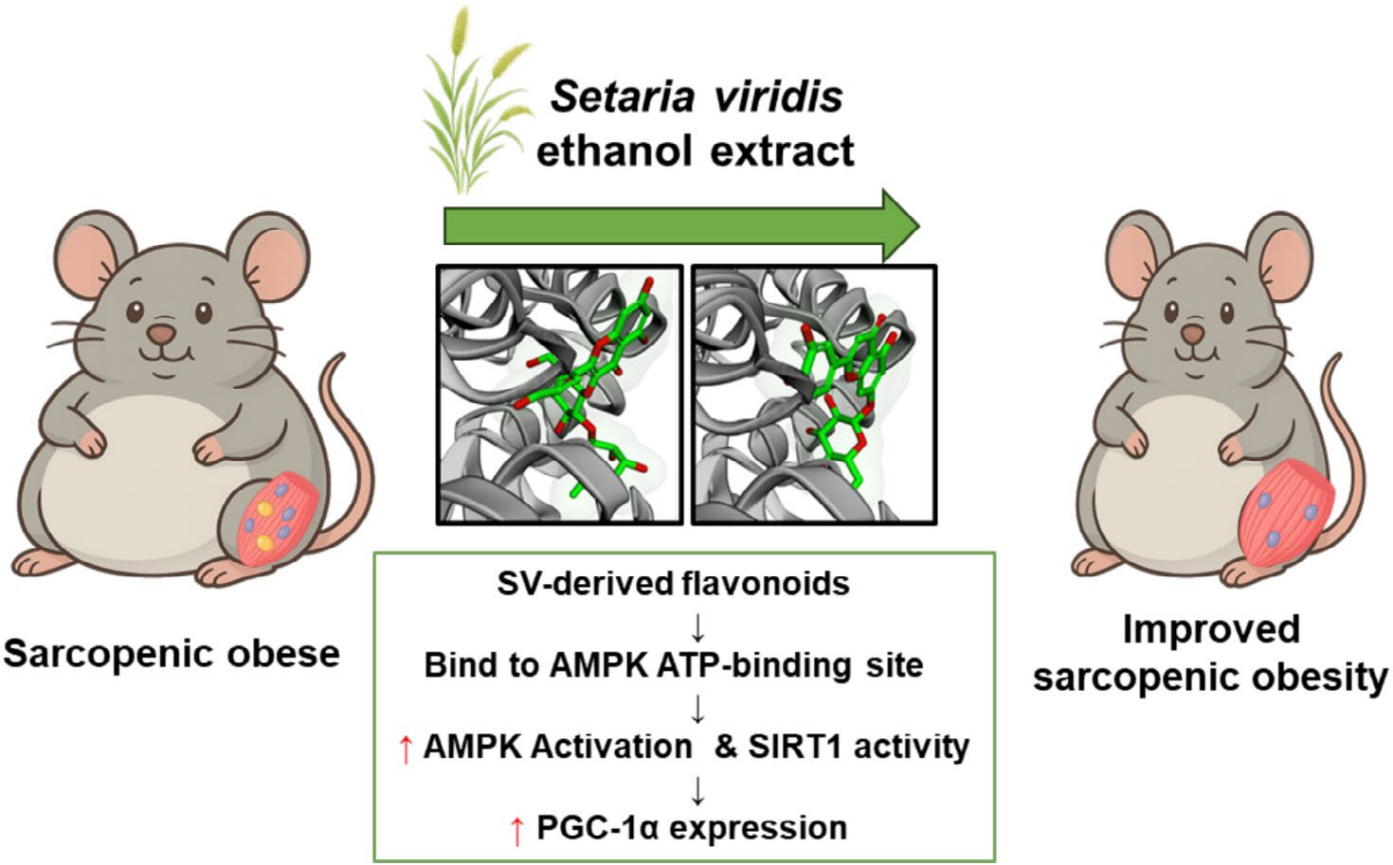
Schematic illustration of the proposed mechanism by which SV‐derived flavonoids activate the AMPK–SIRT1–PGC‐1α signaling pathway to improve muscle metabolism in HFD‐fed mice.

This study elucidated the multifaceted therapeutic benefits of *Setaria viridis* (SV) supplementation in addressing sarcopenic obesity, a condition characterized by the simultaneous presence of excess adiposity and skeletal muscle loss (Figure 6). In HFD‐induced obese mice, SV supplementation led to a significant reduction in fat mass and improvement in muscle mass, morphology, and functional indices. These effects were largely mediated through the activation of AMP‐activated protein kinase (AMPK), which in turn enhanced downstream effectors such as SIRT1 and PGC‐1α, contributing to improved mitochondrial biogenesis, lipid metabolism, and protein synthesis.

Furthermore, SV reduced systemic inflammation and oxidative stress, both of which are key contributors to muscle fibrosis and dysfunction in sarcopenic obesity. These effects were reflected in the suppression of pro‐inflammatory cytokines (e.g., TNF‐α, IL‐6) and the upregulation of antioxidant enzymes, including glutathione (GSH), glutathione reductase (GR), and glutathione peroxidase (GPx).

Notably, 3D molecular docking simulations revealed that SV‐derived flavonoid glycosides—luteolin 7‐O‐glucoside and kaempferol 3‐O‐neohesperidoside—could bind to the ATP‐binding site of AMPK. Among these, luteolin exhibited a stronger binding affinity and greater structural stability, indicating its potential as a primary AMPK‐activating component. In contrast, kaempferol showed weaker binding energy, suggesting a possible supporting role.

Collectively, these findings highlight SV as a promising plant‐derived intervention that targets both metabolic and muscle‐regulatory pathways. Through flavonoid‐mediated AMPK activation, SV supplementation offers therapeutic potential for improving muscle health, mitigating fat accumulation, and enhancing quality of life in individuals with sarcopenic obesity. Further preclinical and clinical studies are warranted to validate these results and assess long‐term safety and efficacy in human populations.

## Author Contributions

Eun‐Young Kwon: conceptualization, funding acquisition, writing – review and editing. Ji‐Won Kim: formal analysis, data curation, writing – review and editing. Ji‐Young Choi: writing – review and editing, funding acquisition. Kyoungho Hwang: data curation and visualization. Youngji Han: data curation, writing – original draft, writing – review and editing, project administration, supervision.

## Conflicts of Interest

The authors declare no conflicts of interest.

## Supporting information


Data S1.

## Data Availability

Research data are not shared.
