# Correction to “*Setaria viridis* Ethanol Extract Attenuates Muscle Loss and Body Fat Reduction in Sarcopenic Obesity by Regulating AMPK in High‐Fat Diet‐Induced Obese Mice”

**DOI:** 10.1002/fsn3.70779

**Published:** 2025-08-18

Kwon, E.‐Y., J.‐W. Kim, J.‐Y. Choi, K. Hwang, and Y. Han. 2025 “*Setaria viridis* Ethanol Extract Attenuates Muscle Loss and Body Fat Reduction in Sarcopenic Obesity by Regulating AMPK in High‐Fat Diet‐Induced Obese Mice.” *Food Science & Nutrition* 13, no. 7: e70655. https://doi.org/10.1002/fsn3.70655

In the article, Figure 4D was mistakenly replaced with a PCR data panel, instead of the intended 3D molecular docking image.

The correct figure is as below:

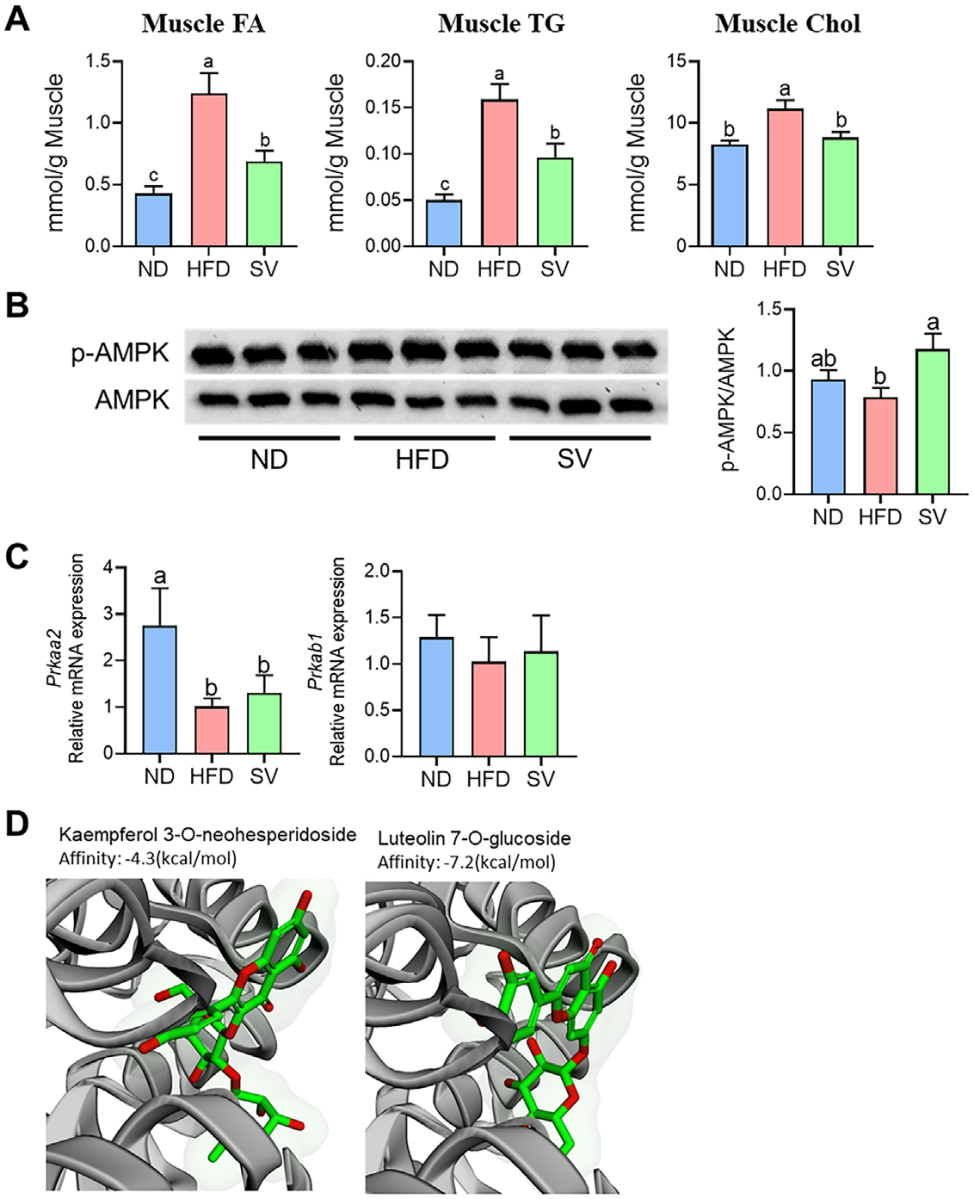

We apologize for this error.

