# Supplementary material for: Setaria viridis Ethanol Extract Attenuates Muscle Loss and Body Fat Reduction in Sarcopenic Obesity by Regulating AMPK in High‐Fat Diet‐Induced Obese Mice
Source: Food Sci Nutr. 2025 Jul 18;13(7):e70655. doi: 10.1002/fsn3.70655 (PMC12274159; doi:10.1002/fsn3.70655)
Supplement: Supplementary file 1 — Data S1. [file FSN3-13-e70655-s001.docx]

Supplementary Table 1. Diet composition for animal experiment

| **Ingredient (g)** | **ND** | **HFD** | **SV** |
| --- | --- | --- | --- |
| Casein | 200 | 265 | 265 |
| Corn Starch | 397.486 | 0 | 0 |
| Sucrose | 100 | 90 | 90 |
| Dextrose | 132 | 160 | 160 |
| Cellulose | 50 | 65.6 | 65.6 |
| Soybean Oil | 70 | 30 | 30 |
| Lard | 0 | 310 | 310 |
| Mineral Mix^1^ | 35 | 48 | 48 |
| Vitamin Mix^2^ | 10 | 21 | 21 |
| Calcium phosphate, Dibasic | 0 | 3.4 | 3.4 |
| TBHQ, antioxidant | 0.014 | 0 | 0 |
| L-Cystine | 3 | 4 | 4 |
| cholin Bitartrate | 2.5 | 3 | 3 |
| SV^3^ |  |  | 3 |
| Total (g) | 1,000 | 1,000 | 1,003 |
| Total energy (kcal) | 4000 | 5,220 | 5,220 |

^1^ AIN-93G- Mineral Mixture (g/kg): calcium carbonate anhydrous, 357; potassium phosphate monobasic, 196; potassium citrate tripotassium monohydrate, 70.78; sodium chloride, 74; potassium sulfate, 46.60; magnesium oxide, 24; ferric citrate, 6.06; zinc carbonate, 1.65; sodium meta-silicate⋅9H_2_O, 1.45; manganous carbonate, 0.63; cupric carbonate, 0.30; chromium potassium sulfate⋅12H_2_O, 0.275; boric acid, 0.0815; sodium fluoride, 0.635; nickel carbonate, 0.0318, lithium chloride, 0.0174, sodium selenite anhydrous, 0.01025; potassium iodate, 0.010; ammonium paramolybdate⋅4H_2_O, 0.00795; ammonium vanadate 0.0066; powdered sucrose, 221.026 ^2^AIN-93G- Vitamin Mixture (g/kg): nicotinic acid, 3; Ca pantothenate, 1.6; pyridoxine-HCl, 0.7; thiamin-HCl, 0.6; riboflavin, 0.6; folic acid, 0.2; biotin, 0.02; vitamin B-12 (cyanocobalamin), 2.5; vitamin E (all-*rac*-α-tocopheryl acetate), 15; vitamin A (all-*trans*-retinyl palmitate), 0.8; vitamin D-3 (cholecalciferol), 0.25; vitamin K-1 (phylloquinone), 0.075; powdered sucrose, 974.655. ^3^SV, *Setaria viridis.* ND, normal diet (AIN-93G, 16% kcal from fat); HFD, high-fat diet (60% kcal from fat); SV, HFD+ *Setaria viridis* ethanol extract (0.3%, *w/w*).

**Supplementary Table S2. Information on antibody in western blot analysis**

| **Antibody** | **Information** |
| --- | --- |
| rabbit anti-mouse *PGC1**-α* | 1:1000; Cell Signaling Technology, Santa Cruz, CA, USA |
| rabbit anti-mouse *α-tubulin* | 1:1000; Cell Signaling Technology, Santa Cruz, CA, USA |
| rabbit anti-mouse *p-Akt* | 1:1000; Cell Signaling Technology, Santa Cruz, CA, USA |
| rabbit anti-mouse *Akt* | 1:1000; Cell Signaling Technology, Santa Cruz, CA, USA |
| rabbit anti-mouse *p-PI3K* | 1:1000; Cell Signaling Technology, Santa Cruz, CA, USA |
| rabbit anti-mouse *PI3K* | 1:1000; Cell Signaling Technology, Santa Cruz, CA, USA |
| rabbit anti-mouse *mTOR* | 1:1000; Cell Signaling Technology, Santa Cruz, CA, USA |
| rabbit anti-mouse *TNF-α* | 1:1000; Cell Signaling Technology, Santa Cruz, CA, USA |
| rabbit anti-mouse *Adiponectin* | 1:1000; Cell Signaling Technology, Santa Cruz, CA, USA |
| rabbit anti-mouse *Haptoglobin* | 1:1000; ABcam, Cambridge, MA, USA |
| rabbit anti-mouse *SCD1* | 1:1000; Cell Signaling Technology, Santa Cruz, CA, USA |
| goat anti-mouse *CerS1* | 1:1000; Novus Biologicals, Littleton, CO, USA |
| rabbit anti-mouse NF-κB | 1:1000; Cell Signaling Technology, Santa Cruz, CA, USA |

**Supplementary Table S3. Predicted binding affinities and docking parameters of SV-derived flavonoid glycosides with AMPK.**

| Kaempferol 3-O-neohesperidoside | | | |
| --- | --- | --- | --- |
| **Mode** | **Binding Affinity (kcal/mol)** | **RMSD l.b. (Å)** | **RMSD u.b. (Å)** |
| 1 | −4.3 | 0 | 0 |
| 2 | −3.2 | 1.779 | 7.847 |
| 3 | −2.4 | 1.887 | 7.724 |
| 4 | −1.5 | 2.756 | 8.094 |
| Luteolin 7-O-glucoside | | | |
| **Mode** | **Binding Affinity (kcal/mol)** | **RMSD l.b. (Å)** | **RMSD u.b. (Å)** |
| 1 | −7.2 | 0 | 0 |
| 2 | −7.2 | 1.905 | 7.853 |
| 3 | −6.9 | 2.479 | 4.073 |
| 4 | −6.7 | 2.608 | 9.448 |
| 5 | −6.6 | 1.486 | 2.321 |
| 6 | −6.6 | 2.191 | 8.818 |
| 7 | −6.3 | 2.413 | 3.784 |
| 8 | −6.3 | 2.210 | 9.192 |
| 9 | −5.9 | 1.738 | 7.803 |

Docking results for kaempferol 3-O-neohesperidoside, luteolin 7-O-glucoside with AMPK predicted by AutoDock Vina. The table lists the top nine docking modes with their corresponding binding affinities (kcal/mol) and RMSD values from the best mode (Å). Mode 1 represents the most favorable conformation with the lowest predicted binding energy.

**Supplementary Figure 1. UPLC-QTOF-MS chromatogram of plant extract (3000 ppm) identifying peaks of Kaempferol and Luteolin glycosides.**


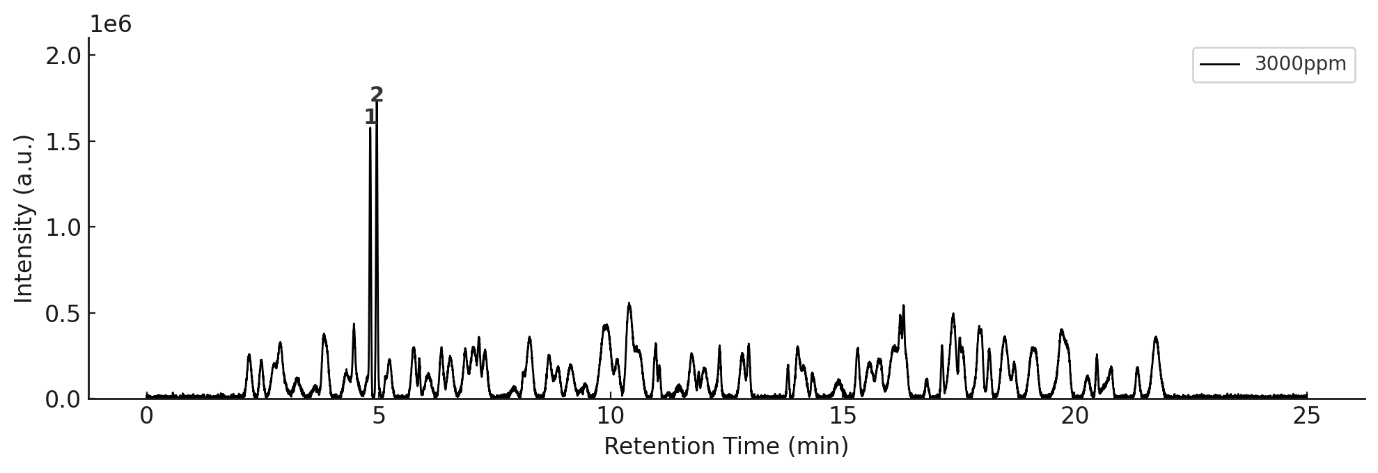


The two numbered peaks correspond to identified flavonoid glycosides: peak 1 at RT 4.82 min represents Kaempferol 3-O-neohesperidoside, and peak 2 at RT 4.96 min corresponds to Luteolin 7-O-glucoside.
